# Inhibitor of Differentiation 1 (Id1) in Cancer and Cancer Therapy

**DOI:** 10.7150/ijms.42805

**Published:** 2020-04-06

**Authors:** Zhengxiao Zhao, Zhiyuan Bo, Weiyi Gong, Yong Guo

**Affiliations:** 1 Department of Oncology, the First Affiliated Hospital of Zhejiang Chinese Medical University, Hangzhou, Zhejiang 310006, China.; 2 The Second Department of Biliary Tract Surgery, Shanghai Eastern Hepatobiliary Surgery Hospital, Shanghai 200438, China; 3 The Department of Integrative Medicine, Huashan Hospital, Fudan University, 12 Middle Urumqi Road, Shanghai 200040, PR China

**Keywords:** Id1, Cancer, Signaling pathway, Angiogenesis, Resistance, Target

## Abstract

The inhibitor of DNA binding (Id) proteins are regulators of cell cycle and cell differentiation. Of all Id family proteins, Id1 is mostly linked to tumorigenesis, cellular senescence as well as cell proliferation and survival. Id1 is a stem cell-like gene more than a classical oncogene. Id1 is overexpressed in numerous types of cancers and exerts its promotion effect to these tumors through different pathways. Briefly, Id1 was found significantly correlated with EMT-related proteins, K-Ras signaling, EGFR signaling, BMP signaling, PI3K/Akt signaling, WNT and SHH signaling, c-Myc signaling, STAT3 signaling, RK1/2 MAPK/Egr1 pathway and TGF-β pathway, etc. Id1 has potent effect on facilitating tumorous angiogenesis and metastasis. Moreover, high expression of Id1 plays a facilitating role in the development of drug resistance, including chemoresistance, radiation resistance and resistance to drugs targeting angiogenesis. However, controversial results were also obtained. Overall, Id1 represent a promising target of anti-tumor therapeutics based on its potent promotion effect to cancer. Numerous drugs were found exerting their anti-tumor function through Id1-related signaling pathways, such as fucoidan, berberine, tetramethylpyrazine, crizotinib, cannabidiol and vinblastine.

## Introduction

Inhibitor of differentiation or DNA binding (Id) was first isolated by Benezra, et al. in 1990 1. Id proteins belong to the helix-loop-helix (HLH) family. Proteins of HLH family contain an HLH dimerization domain which composed of two conserved amphipathic α helices separated by a loop and an adjacent region that contacts DNA 2. Basic HLH (bHLH) proteins bind to a DNA sequence known as an E-box or to the related N-box, which is found in the promoter-enhancer of expressed genes. The formation of heterodimers is an essential procedure for DNA binding and transcriptional activation *in vivo* 3. The bHLH transcription factors are inhibited by class V HLH protein, the Id proteins, which consist of four subtypes, namely Id1, Id2, Id3, and Id4. Id proteins lack a DNA binding domain, and they function as dominant negative regulators of basic HLH transcriptional factors through heterodimerizing with other bHLH factors such as MyoD 1 and E1A 4 and inhibiting their binding to DNA 5 . In humans the four Id genes are located on chromosomes 20q11 (Id1), 2p25 (Id2), 1p36.1 (Id3), and 6p21-p22 (Id4) 6-8 and Id proteins can be found both in the nucleus and in the cytoplasm 9.

Id proteins tightly regulate the expression of cell cycle regulators and orchestrate cell differentiation as well as cell linkage commitment 10, 11. Usually, Id gene expression is positively regulated in undifferentiated, highly proliferative, embryonic or cancer cells, especially for Id1, Id2 and Id3, as Id4 presents distinct functions from Id1, 2, 3 proteins 12. Of all the Id proteins, Id1 is the most extensively studied and mostly linked to tumorigenesis, cellular senescence as well as cell proliferation and survival 13, 14. This review will focus on the research progress of Id1 in the context of cancer and its treatment over the past decade.

## Id1 in cancer-associated pathways

Oncogene is a gene that has the potential to cause cancer. In tumor cells, they are often mutated or overexpressed. Id1 does not strictly meet this classical definition of oncogene, because no tumor-associated mutations in Id1 gene have been observed 15. But Id proteins are overexpressed in over twenty types of cancer including breast cancer, prostate cancer, pancreatic cancer 16, ovarian cancer, endometrial cancer, bladder cancer, melanomas and lung cancer, etc. 17, and it is generally considered as tumor promoter 18. Id1 contributes to tumorigenesis mainly because its role in regulating proliferation and differentiation. Pathways involving Id1 in different types of tumor are described in the following subsections and briefly summarized in Table 1.

### Lung cancer

Id1 promotes carcinogenesis and metastasis, and predicts prognosis of non-small cell lung cancer (NSCLC). Study shows that Id1 and Id3 co-expression is associated with a poor clinical outcome in patients with locally advanced NSCLC 19, 20. In analysis of 532 NSCLC patients' samples, Id1 was found significantly correlated with EMT-related proteins and it enables the tumor and the microenvironment to colonize the liver. Another study of high quality including 457 NSCLC patients also showed the independent prognostic value of Id1 levels for both stage I to stage IV patients that higher Id1 levels were associated with a shorter disease-free survival and overall survival in adenocarcinoma patients 21. Genetic loss of Id1 in the host tissue (Id1-/- mice) impaired liver colonization and increased survival of Id1-/-animals 22. Suppressing both Id1 and Id3 expression was accompanied by decreased angiogenesis and increased apoptosis and greatly reduced the average size of small cell lung cancer in nude mice 23.

In smokers, nicotine binds to pentameric nicotinic acetylcholine receptors (nAChRs) and promotes the growth and metastasis of lung cancers by modulating various signaling cascades, for instance, K-Ras signaling 24, 25. In non-smokers, epidermal growth factor receptor (EGFR) kinase domain mutations which have been established as valid predictors of increased sensitivity to EGFR kinase inhibitors are prevalent in lung cancers 26. Study shows that Id1 gene is a downstream mediator of both K-Ras and EGFR signaling. Both nicotine and epidermal growth factor (EGF) could induce the expression of Id1 in a Src-dependent manner 27. Additionally, BMP signaling in lung cancer cells increases expression of Id proteins and then Id1 regulates lung cancer cell cycle progression by activating CDK4/cyclin D1 and enriching cells at S and G2/M phases so that promotes cell proliferation and growth of lung cancer cells expressing stem cell markers, Oct4 or Nestin 28, 29. Id1 also induces the expression of Stathmin-like3 (STMN3), a microtubule destabilizing protein, and GSPT1, a protein involved in translation termination by down-regulating the expression of two transcriptional co-repressors, NRSF and ZBP89 27, 30 . Furthermore, Akt activation was observed to be involved in cell proliferation-promoting activity of Id1, which was blocked after treating Id1-overexpressing lung cancer cells with PI3K/Akt inhibitor wortmannin 28.

### Glioblastoma

Glioblastoma (GBM) is grade IV glioma which is characterized by poor therapeutic response and poor overall survival. Differentiation therapy has been proposed as a promising strategy for GBM therapy, as upon differentiation, GBM cells lose tumorigenicity and become sensitive to chemotherapy and radiotherapy 31. Id1 serves as a main mediator that abrogates differentiation signals in glioblastoma stem cells (GSCs) and contributes to GBM initiation and chemoresistance in GBM. Knocking out of Id1 in GBM reduced tumor progression 32. Study shows that Id1 can simultaneously regulates stemness through WNT/SHH signaling and differentiation through bone morphogenetic protein receptor(BMPR)-mediated differentiation signaling in GSCs. Id1 activates WNT and SHH signaling by upregulating Dvl2 and Gli2 proteins through suppression of Cullin3 E3 ubiquitin ligase in a ligand-independent manner 33. Activation of WNT and SHH signaling increases the expression of Myc and its transcriptional targets miR-17 and miR-20a, then the two miRNAs inhibit expression of the differentiation-inducing receptor, BMPR2 34. In GBM cells, Id1 is also a potential downstream effector of protein tyrosine kinase 7(PTK7) and transglutaminase 2 (TGM2) which are highly expressed in CD44-high glioblastoma and predicts unfavorable prognosis. Depletion of PTK7 or TGM2 inhibitor treatment reduced Id1 expression and overexpression of Id1 mostly restored the cell proliferation and colony formation 35, 36. Id1 is regulated by ERK1/2 MAPK/Egr1 pathway and TGF-β pathway as well, activation of these pathways increases self-renewal capacity of GBM cells and resistance to radiation-induced DNA damage 37, 38.

### Leukemia

Leukemia is a malignant hematological disorder characterized with different clinical manifestations, cellular and molecular mechanisms, and different response to therapy or risk of relapse. Overexpression of Id1 is seen in acute myeloid leukemia (AML) patients. Studies showed that high expression of Id1 is associated with poor prognosis in patients with AML, independently predicting shorter disease-free survival and overall survival 39, 40, especially for those with higher risk karyotype classification in young non-M3 patients 41. Noticeably, Id1 expression is not an independent prognostic factor in acute myeloid leukaemia with normal karyotype when CEBPA mutations were included in the analysis 42. Study suggests that Id1 is a key transcriptional regulator of hematopoietic stem cell lineage commitment and it can immortalize hematopoietic progenitors* in vitro* and promote a myeloproliferative disease in mice *in vivo* 14, 43. Oncogenic tyrosine kinases, such as BCR-ABL, TEL-ABL, TEL-PDGF beta R, and FLT3-ITD, play a major role in the development of hematopoietic malignancy. Id1 was identified as a common downstream target of constitutively activated oncogenic tyrosine kinases 44. Furthermore, loss of Id1 inhibited t(8;21) leukemia initiation and progression by abrogating AKT1 activation 45. It is different from other tumors that the differential role of Id1 in MLL-AF9-driven leukemia is basing on cell of origin. Study shows that mice receiving MLL-AF9-transduced fetal liver cells or bone marrow cells develop AML. Loss of Id1 significantly prolonged the median survival of mice receiving fetal liver cells but accelerated leukemogenesis in recipients of bone marrow cells 46. The effect of Id1 in leukemogenesis seems largely dependent on p21. The expression of p21 is extremely low in human fetal hematopoietic stem/progenitor cells, but it increases as the cells differentiate into myeloid cells 46, 47. Conclusion was also gained by researchers that low expression of Id1 was observed in most AML cell lines and human AML samples 48. This indicates that Id1 expression in AML is quite different from that reported in other human malignancies where the Id1 expression is up-regulated. But small sample size limited its reliability.

### Breast cancer

High Id1 expression in breast cancer cell lines is associated with high aggressiveness and metastasis which is a major factor responsible for mortality in patients with breast cancer 15, 49. Id1 induces mammary tumorigenesis by increasing normal and malignant mammary stem cell activities in transgenic mice 50. Id1 also induces metastatic mammary carcinoma by cooperating with oncogenic Ras 51. Higher Id1 expression was associated with worse disease-free survival and overall survival 52.. Targeting Id1 expression in breast cancer cells reduces breast cancer metastasis in animal models. Breast tumors failed to grow and metastasize in Id1 (+/-) Id3 (-/-) mice. Mern, D.S., et al isolated a novel peptide aptamer, d1/3-PA7(LSAMAATLFAELGCHLSRWM), specifically interacting with Id1 and Id3 from randomized combinatorial expression library using yeast and mammalian two-hybrid systems. It can significantly provoke anti-proliferative and apoptotic effects in breast cancer cells 53. Gurrapu, S et al. suggests that Semaphorin 4C reverse signaling sustains the metastasis formation through induction of prometastatic genes including Id1 and Id3 54. Study indicated that Id1 promotes breast cancer metastasis by S100A9 regulation. Id1 interacts with TFAP2A to suppress S100A9 expression. Migratory, invasive phenotypes in vitro and metastasis in vivo induced by Id1 expression can be rescued by reestablishment of S100A9 expression 55. Id1 gene expression was also correlated with EMT-associated genes including, VIM, SNAI1, SNAI2, and TWIST1 56. Both ERβ1, a member of the nuclear receptor superfamily of ligand-regulated transcription factors and an important protein in regulating the progression of breast cancer 57 , and CCND1, a regulator of cyclin dependent kinase, regulate the migration and invasion of breast cancer cells in an Id1-dependent manner 56. Abl interactor 1 (Abi1) is a critical regulator of actin polymerization/depolymerization, involving in the abnormal development of cytoskeletal functions of breast cancer cells. Its regulating function of the invadopodia formation depends on the Id1, as well 58. Furthermore, addiction to the IGF2-Id1-IGF2 circuit is essential for maintenance of the breast cancer stem-like cells 59. But controversial conclusion was also gained, Zhou XL et al. showed increased Id1 mRNA levels were associated with higher relapse‑free survival rates in all patients with breast cancer through analysis of data from a set of publicly accessible databases 60. Even in a same study, paradoxical results were also acquired. Gurrapu, S et al. found that semaphorin 4C elicited Id1/3 dependent metastasis of breast cancer and prostate cancer, but in culture, they found that semaphorin 4C overexpression impaired cancer cell migration and invasivenesss 54. This means the effect of Id1 in the development of cancer is complicated, its role in the primary foci of cancer, the shedding and colonization of cancer cells, and the metastatic cancer needs further studied.

### Prostate cancer

In mouse prostate model, overexpression of Id1 alone is not sufficient to drive neoplastic change 61, but Id1 is proved to regulate proliferation, apoptosis, and androgen-independence of prostate cancer (PCa) cell. Increased Id1 protein expression is strongly associated with increasing grade of PCa 62. And a study involving 52 prostate cancer patients showed higher Id1 RNA expression predicted a higher hazard ratio for progression and a shorter disease-free survival 63. Knocking out Id1 gene has an in-vivo preventive effect against the development of prostate cancer in mouse model 64. Id1 reduction is pre-requisite for inhibitory effects of TGFβ on cell proliferation and migration. Cross talk with MAPK, NFκB and TNFα promotes cell survival, proliferation, metastasis and androgen-independence 65, 66. Mechanism is related to attenuation of all three cyclin-dependent kinase inhibitors (Cdkn2b, -1a, and -1b) by increased Id1 expression 62. Id1 induced immortalization was also associated with decreased expression of Cdkn2a, Cdkn1a, androgen receptor (AR) and increased Tert expression. Network analysis indicates that Id1 promotes cancer morphology, cell cycle and epithelial to mesenchymal transition by influencing AP1, tnf, tgfbeta, PdgfBB and estradiol pathways 66. Intriguingly, study also showed that Id1 could down-regulate the ability of PC3 cells to form osteolytic lesions in vivo, although in the same study it was observed that knockdown of Id1 in PC3 cells inhibited the proliferation of cancer cells in vitro 67. While evidence also suggests that Id1 is a key factor in promoting cancer metastasis to lungs 68, the discrepancy of microenvironment in different sites may be the cause. Moreover, Id1 was showed mediating chemosensitivity enhancement. Patients with higher Id1 expression were found to be associated with longer relapse-free survival than patients without Id1 increase after neoadjuvant chemotherapy and radical prostatectomy. What's more, in the prostate cancer cell line LNCaP, docetaxel dose-dependently induced Id1 transcription and stable Id1 overexpression in LNCaP enhanced docetaxel-induced cytotoxicity 69. This means the role of Id1 in prostate cancer is complicated and should be analyzed in specific.

### Cervical cancer

Human papillomavirus (HPV) infection is tightly associated with cervical cancer. High expression of Id1 protein was found to be correlated with E6 oncoprotein in high-risk HPV and HPV-immortalized cervical epithelial cells which suggested that Id1 plays an oncogenic role in HPV-related cervical carcinogenesis 70-72. Study shows that Id1 expression is an independent prognostic marker in early-stage cervical cancer, patients with strong or moderate expression of Id1 have a significant shorter overall survival time 73. Co-expression of Id1 and nuclear NF-κB p65 promotes progression and malignancy of cervical cancer 74. Moreover, inflammation exerts prominent function in tumorigenesis of cancer. A 37 kDa protein, annexin A1 (ANXA1), was found to be an anti-inflammatory mediator and expressed by tumor cells. Study showed that ANXA1 down-regulated Id1 gene expression and the Id1 pathway gene, BMPR1B, in cervical cancer 75. This indicates that Id1 also plays a role in tumor-associated inflammation.

### Thyroid cancer

In human thyroid tissue, Id1 protein expression increases gradually from normal thyroid tissue, hyperplastic thyroid tissue to malignant thyroid tissue 76. But no significant association between Id1 protein expression level and tumor-node-metastasis stage, tumor size, primary tumor vs. lymph node metastasis, primary tumor vs. recurrent tumors, and extent of tumor differentiation was found which means Id1 is not a marker of aggressive phenotype in differentiated thyroid cancer 77. With regard to the association between Id1 level and overall survival or disease free survival for thyroid cancer patients needs to be further studied. Id1 gene expression was induced by many growth factors in various cell lines, including TGFβ1, PDGF, NGF, EGF, IGF-1, and estrogen 78. In thyroid cancer the Id1 mRNA expression was upregulated by thyroid-stimulating hormone (TSH) 79. And Id1 protein has been found to be an early target of TGFβ, it induces mesenchymal phenotype and promotes invasiveness of thyroid tumor cells 80.

### Colorectal cancer

Higher Id1 expression in colorectal cancer specimens than in normal mucosal specimens was shown and high Id1 expression positively correlated with poor differentiation in colorectal cancer 81. Knock down of Id1 arrests the growth of colorectal cancer cells and suppressed hepatic metastasis in vivo 82, except for colitis-associated colorectal cancer 83. Downregulation of PCNA, survivin, CXCR4, MMP2 and MMP9 was found in Id1 knock down cells 82. Id1 maintains the stemness of colorectal cancer cells through the Id1-c-Myc-PLAC8 axis through activating the Wnt/β-catenin and Shh signaling pathways 81. Self-renewal and metastatic colonization of tumor-initiating cells in colorectal cancer were regulated by miR-371∼373/TGFβ receptor 2/Id1 signaling axis and p21/Id1 pathway 84, 85. Study also showed cell division cycle protein 27 (CDC27) mutation promoted metastasis and sphere-formation capacity of colorectal cancer cells in an Id1-dependent manner 86. In addition, p53/stat3/Id1 pathway mediates chemotherapeutic resistance of colorectal cancer 87.

### Hepatocellular cancer (HCC)

Id1 is relevant to HCC dedifferentiation 88, Id1 levels are not only high in HCC cells, but also upregulated in HCV-infected hepatic cells or viral core gene-transfected cells, whereas they are very low in normal liver tissues 89. Moreover, overexpressed Id1 is associated with patients' prognosis and HBx expression in hepatitis B virus-related HCC. It contributes to the development of HCC with cirrhosis 90 and is a potential prognostic marker for HBV-related HCC 91. Patients with overexpression of Id1 had shorter disease-free and overall survival times 92. Indeed, Id1 promotes metabolic reprogramming in HCC cells 93. AR activity is associated with cancer development and progression. In HCC, AR contributes to the incidence of HCC. AR activation enhanced the expression of Id1, which led to increased HCC cell migration and invasion 94. Study found that Id1 is mediated by the MAPK/ERK pathway and associated with increased c-Myc levels in HCC. Id1 knockdown leads to c-Myc reduction as well as c-Myc knockdown leads to Id1 reduction. Moreover, Id1 may interact directly with c-Myc without inhibiting the transcriptional activity of c-Myc 93. However, a totally adverse conclusion was also acquired in other studies. Lei-lei Niu, et al. showed that Id1 protein was down-regulated in 15 out 20 HCC tumors compared to matched non-tumor tissues (even though the title of this paper is improper) 95. Damdinsuren B, et al. reported Id1 protein was highly expressed in non-tumor liver tissues with hepatitis and cirrhosis. The decreased expression of Id1 was observed in 372 liver HCC samples, compared to adjacent normal samples 88.

## Id1 in tumor angiogenesis and metastasis

Sufficient nutrient supply guaranteed by new blood vessels is crucial to tumor progression and metastasis. Tumor angiogenesis is significantly triggered by the upregulation of vascular endothelial growth factor (VEGF). Study showed VEGF-A expression is regulated by TGF-β1 through Id1 pathway 96. Numerous studies indicated the important role of Id1 in angiogenesis, for example, in small cell lung cancer, suppressed expression of Id1 and Id3 was accompanied by decreased angiogenesis 23. Id1+/- Id3-/- mice fail to grow tumors due to poor vascularization and necrosis 97. MiR-885-3p downregulates Id1 by targeting BMPR1A, leading to impaired angiogenesis 98. High expressions of Id1 and matrix metalloproteinase 9 (MMP9) have tight correlations with the development and progression of colorectal adenocarcinoma and have positive correlations with microvascular density. Both of them may be involved in the microvascular generation, the invasion and hematogenous metastasis of colorectal carcinoma 99. Membrane degradation and cell migration were partly mediated by matrix metalloproteinases (MMPs). Id1 can increase MMP gene expression, leading to tumor cell invasion. High levels of Id1 and the membrane-type 1-MMP (MT1-MMP) or MMP1 were associated to breast cancer metastasis 100, 101. KLF17 is a zinc-finger protein acting as a metastasis suppressor. It can inhibit Id1 transcription through binding to its promoter region. KLF17 is significantly down-regulated in primary human breast cancer samples, thus leading to Id1 induction, which may promote primary tumor vascularization via VEGF production, breast cancer cell invasion and EMT 102.

Phenotypic plasticity, the epithelia-to-mesenchymal and mesenchymal-to-epithelial transition (EMT-MET) switch in particular, is required for cancer metastasis 103. Castañón E et al. analyzed samples of 532 NSCLC patients, they found Id1 significantly correlated with vimentin and other EMT-related proteins. The loss of Id1 decreased the levels of vimentin, integrinβ1, TGFβ1 and snail, both* in vitro* and *in vivo*. In their study, Id1 facilitated lung cancer liver colonization through activation of EMT program in tumor cells and establishment of the pre-metastatic niche 22. In breast cancer, Id1 induced by TGF-β opposes Twist1 and promotes metastatic colonization to lung via EMT 104. E47 protein (encoded by E2A gene) is a member of the class I bHLH transcription factors (also known as E protein). E47 has been described as a repressor of E-cadherin and inducer of EMT. Study found that E47 interacts with Id1 in E47 overexpressing MDCK cells that underwent EMT as well as in mesenchymal breast carcinoma and melanoma cell lines 105.

## Id1 in therapeutic resistance

Numerous therapeutic methods were applied in anti-cancer treatment, such as chemotherapy, radiotherapy, targeted therapy and immunotherapy. But drug resistance which leads to a more aggressive cancer and poor prognosis is a severe limitation. Mechanism is associated with a sub-population of tumor cells with stem-like properties, cancer stem cells (CSCs) which are specifically endowed to resist or adapt to the standard therapies, leading to therapeutic resistance. Based on the fact that Id1 serves as a stem cell-like gene and knockdown of Id1 suppresses the expression of the key CSC-associated factors Nanog and octamer-binding protein 4 (Oct-4), a role of Id1 in the development of drug resistance has been suggested 106. Study demonstrated that knockdown of Id1 sensitized gastric cancer cells to cisplatin 106. miR381 interfered with NF-κB through repression of Id1 and thus re-sensitized A549/CDDP cells to cisplatin. Co-expression of Id1 reversed the enhancement of cisplatin cytotoxicity by miR-381 107. In NSCLC, Id1 and Id3 co-expression is associated with a poor clinical outcome in patients 19. High Id1 expression is a negative prognostic factor. However, it paradoxically predicts a favorable prognosis for adjuvant paclitaxel plus cisplatin therapy in surgically treated lung cancer patients 108. And Id1 overexpression increases gefitinib sensitivity in NSCLC, regardless of the mutational status of NSCLC. Mechanism is related to activation of RIP1/RIP3/MLKL pathway dependent necroptosis 109. In HCC, Id1 knockdown activates p16/IL6 axis and contributes to the resistance of HCC to sorafenib 95. Id1- induced pentose phosphate pathway activation confers chemoresistance to oxaliplatin and promotes HCC proliferation 110. In colorectal cancer, after long time 5-Fu selection, Id1 expression was top upregulated in colorectal cancer cells and more aggressive tumors was generated 111. Decreasing the expression of Id1 gene was able to restore the sensitivity to 5-Fluorouracil (5-FU) 112, 113. Mechanistically, study showed that Id1 conferred 5-FU chemoresistance through E2F1-dependent induction of thymidylate synthase expression 114. In GBM multiforme, Id1 induced by cyclooxygenase-2 (Cox-2)-derived prostaglandin E2 (PGE2) increases GBM self-renewal and radiation resistance. Mechanism was found to be via EP4-dependent activation of MAPK signaling and the Egr1 transcription factor 115. And the inhibition of Id1 enhances the effect of temozolomide, delays tumor recurrence, and prolongs survival 32. But an opposite conclusion was also acquired by Guo, Q et al. who found that GBM patients with high Id1 expression had better survival than patients with low Id1 expression since Id1 expression could increase the radiotherapy efficacy 116. Same conclusion was also obtained in a study of prostate cancer. They found stable Id1 overexpression in prostate cancer cell line LNCaP enhanced docetacel-induced cytotoxicity and patients with Id1 upregulation possess longer relapse-free survival than patients without Id1 increase 116.

**Figure 1:**
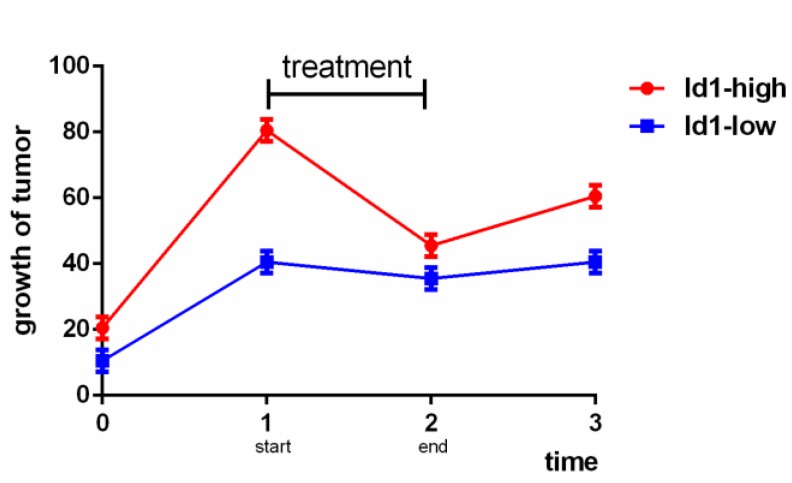
Analog trend graph of relationships among Id1, therapy and growth of tumor.

According to the above, we know that the role of Id1 in therapeutic resistance varies as cancer type varies. Mostly, Id1 is hazardous to the therapeutic resistance and prognosis of cancer, but in NSCLC, GBM and prostate cancer some shows that it is a sensitive marker to chemotherapy or radiotherapy. However, besides cancer types, the controversial conclusion may also related to the concrete expression amount of Id1, the duration of treatment, the observation time and specific medicine. Studies provided more details should be commenced and the balance between Id1 and treatment should be stressed because the relationships among Id1 expression, therapy and growth of tumor may present like Fig 1 below.

## Id1 as therapeutic targets

Regarding to low survival rate of patients with cancer, researches of novel, potent anti-tumor therapeutics are essential. The Id1 represent an interesting target for this purpose, as it is involved in cellular key events related to tumorigenesis and cancer progression 117. Anti-proliferative and chemosensitization effect of gamma-tocotrienol on breast cancer cells was mediated through downregulation of Id1 protein 118. HCC invasion was suppressed by fucoidan treatment both* in vitro* and* in vivo* which was related to NDRG-1/CAP43-dependent down-regulation of Id1 119. Berberine also suppressed the growth and development of lung metastases in HCC by inhibiting the expression of Id1. Berberine's anti-proliferative and anti-invasive activities could be partly rescued by Id1 overexpression 120. Tetramethylpyrazine inhibited the growth of lung cancer through disrupting angiogenesis via BMP/Smad/Id-1 signaling pathway 121. Crizotinib decreased Id1 levels in ALK- and MET-positive lung cancer cells and inhibited cell migration 122. Cannabidiol (CBD), a non-toxic, non-psychoactive cannabinoid and redox modulator, could inhibit GSCs survival, self-renewal and significantly increase the survival of GSC-bearing mice by activating p38 pathway and downregulating key stem cell regulators Sox2, Id1 and p-STAT3 123. Furthermore, vinblastine (VBL), a key microtubule inhibitor, was also confirmed that it downregulated Id1 in VBL-treated human cervical carcinoma cells 124. As to the therapy targeting Id1 in leukemia, pimozide, a known USP1 inhibitor was proved effective in inhibiting the growth of primary AML patient-derived leukemic cells 125. USP1 is a deubiquitinating enzyme, which removes polyubiquitin chains from the Id1 protein 126. Many other compounds exert their anti-tumor function via Id1-related signaling pathways or are found possessing effective regulation on Id1.

## Conclusion

Id1 is a member of the HLH family which serves as a regulator of cell differentiation and cell linkage commitment. Generally, it is overexpressed in over twenty types of cancer and promotes growth and metastasis of cancer. Id1 potently induces angiogenesis and EMT. Its role in drug resistance is controversial, whereas most of studies suggested that Id1 is responsible for chemoresistance and radiation resistance. Id1 is a promising target of anti-tumor treatment as many compounds exert anti-tumor properties by mediating Id1-related pathways. Nevertheless, aiming to understand and solve controversial data, to answer open questions and to further validate Id1 as a therapeutic target of cancer and develop new drugs, more work should be done to explore the biological characteristics of Id1 and its relating signaling network.

## Figures and Tables

**Table 1:**
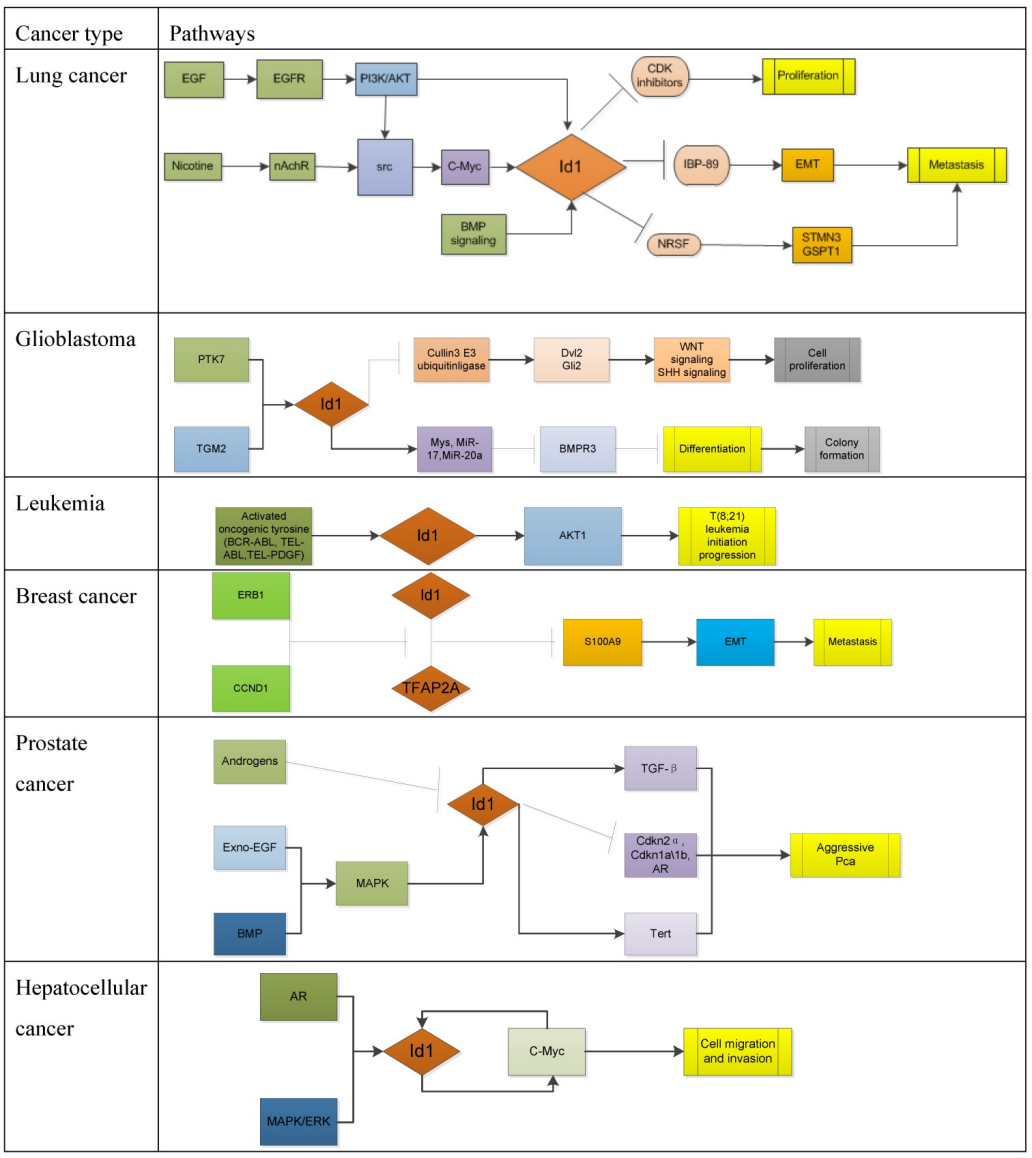
Id1 in different cancer-associated pathways

## Abbreviations

Id1: inhibitor of differentiation 1
HLH: helix-loop-helix
EMT: epithelial-mesenchymal transition
bHLH: basic HLH
NSCLC: non-small cell lung cancer
nAChRs: nicotinic acetylcholine receptors
EGFR: epidermal growth factor receptor
EGF: epidermal growth factor
STMN3: stathmin-like3
GBM: glioblastoma
GSCs: glioblastoma stem cells
BMPR: bone morphogenetic protein receptor
PTK7: protein tyrosine kinase 7
TGM2: transglutaminase 2
AML: acute myeloid leukemia
PCa: prostate cancer
HPV: human papillomavirus
ANXA1: annexin A1
TSH: thyroid-stimulating hormone
CDC27: cycle protein 27
HCC: hepatocellular cancer
AR: androgen receptor
VEGF: vascular endothelial growth factor
MMP9: matrix metalloproteinase 9
MMPs: matrix metalloproteinases
MT1-MMP: membrane-type 1-MMP
EMT-MET: epithelia-to-mesenchymal and mesenchymal-to-epithelial transition switch
CSCs: cancer stem cells
Oct-4: octamer-binding protein 4
5-FU: 5-fluorouracil
Cox-2: cyclooxygenase-2
PGE2: prostaglandin E2
CBD: Cannabidiol
VBL: vinblastine
